# Enhancing the Therapeutic Potential of Mesenchymal Stem Cells with Light-Emitting Diode: Implications and Molecular Mechanisms

**DOI:** 10.1155/2021/6663539

**Published:** 2021-02-03

**Authors:** Barbara Sampaio Dias Martins Mansano, Vitor Pocani da Rocha, Ednei Luiz Antonio, Daniele Fernanda Peron, Rafael do Nascimento de Lima, Paulo Jose Ferreira Tucci, Andrey Jorge Serra

**Affiliations:** ^1^Biophotonics Applied to Health Science, Nove de Julho University, São Paulo, SP, Brazil; ^2^Department of Medicine, Cardiology Division, Federal University of São Paulo, SP, Brazil

## Abstract

This study evaluated the effects of light-emitting diode (LED) on mesenchymal stem cells (MSCs). An electronic search was conducted in PubMed/MEDLINE, Scopus, and Web of Science database for articles published from 1980 to February 2020. Ten articles met the search criteria and were included in this review. The risk of bias was evaluated to report quality, safety, and environmental standards. MSCs were derived from adipose tissue, bone marrow, dental pulp, gingiva, and umbilical cord. Protocols for cellular irradiation used red and blue light spectrum with variations of the parameters. The LED has been shown to induce greater cellular viability, proliferation, differentiation, and secretion of growth factors. The set of information available leads to proposing a complex signaling cascade for the action of photobiomodulation, including angiogenic factors, singlet oxygen, mitogen-activated protein kinase/extracellular signal-regulated protein kinase, Janus kinase/signal transducer, and reactive oxygen species. In conclusion, although our results suggest that LED can boost MSCs, a nonuniformity in the experimental protocol, bias, and the limited number of studies reduces the power of systematic review. Further research is essential to find the optimal LED irradiation parameters to boost MSCs function and evaluate its impact in the clinical setting.

## 1. Introduction

Photobiomodulation (PBM) employs nonionizing forms of light sources, which encompass laser and light-emitting diode (LED) for a broad visible and infrared spectrum, and PBM-based therapy has been successfully applied in treatment of several diseases, injuries, and disorders [1]. Acquaintance with the mechanisms subjacent the effects of PBM has been of considerable interest, and a well-accepted view is that the light energy delivered to tissues is absorbed by the cell chromophores, favouring the production of adenosine triphosphate (ATP) [2, 3]. Nevertheless, beneficial effects on inflammation, oxidative stress, survival, and the regeneration of tissues have been reported by several researchers [4–7].

Concerning LED, when the first one was developed in 1998 by Harry Whelan and his group at the NASA space medicine laboratory [8], this technology had some characteristics that diverge them from laser devices. While LED are noncoherent and quasimonochromatic light sources [9] based on the phenomenon of electroluminescence of semiconductor materials [10], laser emits, in a stimulated manner, a monochromatic, and coherent light beam of low divergence [11]. Besides that, it is important to quote that those different properties would still photoactivate cells without causing heat changes or damage. Notwithstanding, benefits are perceptible in LED when compared to lasers, such as increased safety and durability, lower cost, ease of use, and more flexibility in the irradiated area size [11, 12], although the biological effects of light on irradiated cells are similar in both laser and LED [13, 14]. LED phototherapy has either been well-proven to have an effective benefit in a wide variety of clinical indications such as pain relief, skin injuries, rheumatological diseases, muscle disorders, and infections, suggesting as well that LED might have a powerful role to play in the clinical practice for a variety of conditions. [7, 15–27].

The LED has also emerged to have important effects on mesenchymal stem cells (MSCs) [28], easily cultivated multipotent stem cells which can be isolated from various adult tissues, therefore opening a new window for PBM application into strategies being currently pursued to improve therapy with MSCs. Among the advantages of MSCs usage, it is possible to observe the availability in ubiquitous sources, the extensive ability of proliferation and multilineage differentiation, easy isolation, low immunogenicity, and paracrine potential [29, 30]. In addition, it is important to cite the immunomodulatory proprieties of MSCs, which could be used in the treatment of many disorders like promoting maintenance of the ratio of Treg and T helper cells in systemic lupus erythematosus [31], and the proosteogenic capacity, mainly presented in oral tissue MSCs [31, 32].

MSCs reside in a complex microenvironment among other cell types and biochemical stimuli, which influence if the cell will differentiate or self-renew. Mechanical factors are also being recognized as regulators. Therefore, the microenvironment is significantly an influencer of the role and differentiation of MSCs through biochemical, biomechanical, and biophysical factors [33].

Although being a promising therapy, mostly to the poor engrafting of implanted cells, low survival rates are common for long periods and MSC therapy has generated unsatisfactory results in numerous clinical conditions [34, 35]. Hence, since LED was referred to have the biological effects mentioned above, it could be applied to overcome the current limitations of MSC therapy more easily. Thereby, preconditioning of MSCs with LEDs before transplantation may be a usual procedure to improve tissue engineering and cell therapy in the future [36, 37].

To the best of our knowledge, most of the articles evaluated the repercussion of PBM on MSCs, as illustrated in different systematic reviews [28, 38–40], and the MSC response to LED irradiation remains unclear. Therefore, based on the plethora of biology actions that make the MSCs promising to regenerative medicine [29] and the possible cytoprotective effect of the PBM, we aimed this systematic review to summarize the current evidence about the effects of LED in mesenchymal stem cells (MSCs) and to identify the underlying mechanisms found to underpin this effect.

## 2. Materials and Methods

### 2.1. Search Strategy

**Figure 1:**
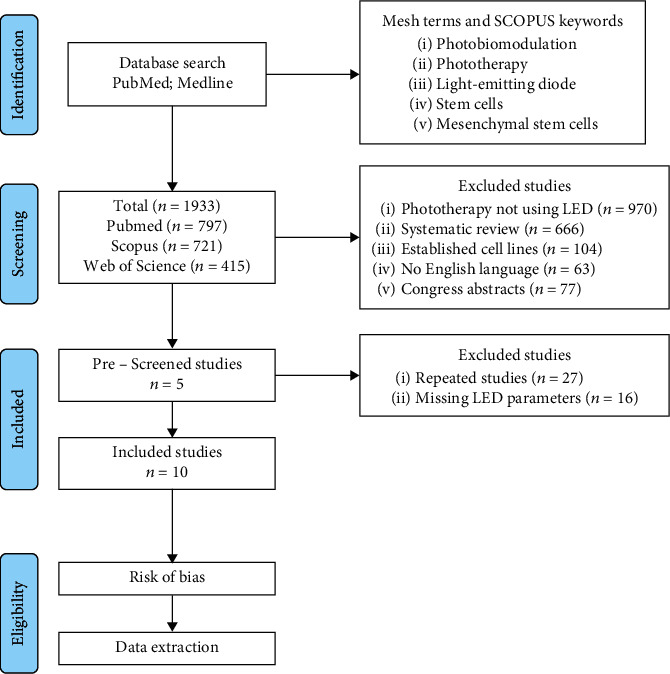
Flow chart diagram for selection of studies.

The study was carried out according to PRISMA guidelines (Figure 1). The search for published articles into the effect of LEDs on MSCs was conducted in PubMed, Scopus, and Web of Science databases. The articles retrieved were limited to the English language and were for the period from January 1980 to February 2020. The MeSH terms and Scopus international data lines were used to find keywords related to “photobiomodulation,” “phototherapy,” “light-emitting diode,” “stem cells,” and “mesenchymal stem cells.” MeSH terms were used individually or combined to increase the findings. Data extraction involved MSC experimental methodology, LED parameters, and results. Duplicate articles from the database search results were removed.

### 2.2. Study Selection

Screening for potentially eligible studies was examined by considering the title and abstracts close to the keywords regarding the theme. Additionally, two independent reviewers applied predetermined inclusion criteria to full studies. Conflicts were solved by a third independent researcher. Articles investigating *in vitro* procedural or methodological applications of LED were accepted. The application of the irradiation could be to any plate or culture bottle. At the end of the selection process, after reading the full texts, articles that matched inclusion criteria are included: a quantitative or semi-quantitative measure; English language; LED irradiation provided as an intervention to at least one of the treatment groups; MSCs experimentally analyzed; to report a minimum of LED parameters; or the missing parameter had to be calculable using alternate parameters. The exclusion criteria were as follows: established cell lines; missing LED parameters or not possible to calculate; papers not published in the English language; phototherapy not using LED; and review articles with or without meta-analysis. Figure 1 shows the process of study selection.

### 2.3. Risk of Bias

Potentially eligible articles were printed, reviewed, and critically judged by three independent reviewers. Bias is a reliable method to assess quality, safety, and environmental standards of clinical and experimental studies [41]; therefore, the studies were analyzed using an adapted version for cellular research [42]. Risk of bias included selection (systematic differences in the comparison groups), performance (systematic differences introduced during the study), detection (systematic differences in the outcome assessment between groups), attrition (systematic differences in excluding study units between groups), reporting (systematic omission of results in the study documentation/publication), confounding (systematic differences in factors potentially influencing the results between groups), appropriate statistical methods, and other bias.

## 3. Results

### 3.1. Study Selection

Figure 1 shows a flowchart of the search, conducted using the three databases. Overall, 1933 articles were found in an early screening. From the initial potentially relevant articles identified, 1880 were excluded because they did not meet the inclusion criteria as follows: no LED irradiation (*n* = 970); systematic reviews (*n* = 666); established cell lines (*n* = 104); no English language (*n* = 63); and congress abstracts (*n* = 77). After this, 53 articles remained, and they were fully examined to check closely the exclusion criteria. Then, 27 repeated studies were excluded as well as 16 other papers with lost or impossible to calculate LED parameters. Finally, 10 articles served as the basis for this systematic review.

### 3.2. Risk of Bias

**Figure 2:**
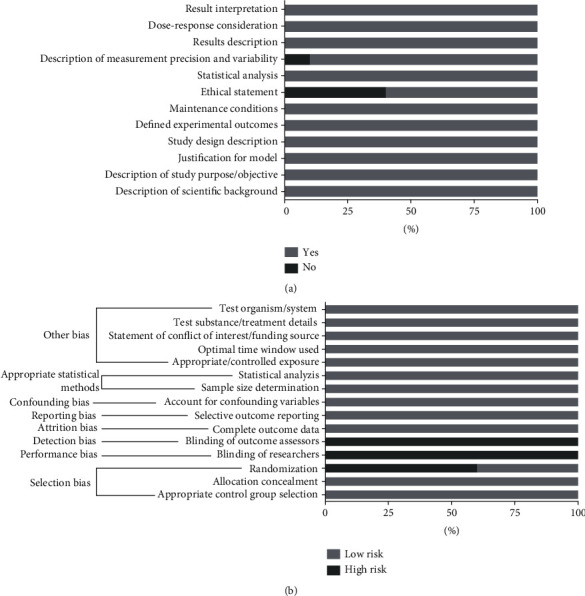
(a) Risk of bias summary: review authors' judgements about each risk of bias item for each included study. (b) Risk of bias graph: review authors' judgements about each risk of bias item presented as percentages across all included studies.

Figure 2(a) illustrates the risk of bias evaluated for each included study. Firstly, articles were surveyed for the presence or absence of key sections: 40% did not mention ethical statement and 10% did not give a description of measurement precision and variability. Then, low and high risk of bias was judged as illustrated in Figure 2(b), in which the categories that presented more studies (i.e., 100%) in low risk were as follows: other bias; appropriate statistical methods; confounding bias; reporting bias; and selection bias—“allocation concealment” and “appropriate control group selection.” Some categories expressed 100% concerning high risk: detection and performance bias. Selection bias (“randomization”) was 60% at high risk.

### 3.3. Interventions

Table 1 describes in detail the methodology of MSCs used in seven articles. MSCs were derived from two animal species (60% human, 40% rodents) and several sources (30% bone marrow, 30% umbilical cord, 20% tooth pulp, 10% adipocyte, and 10% gingiva). Cell concentration and passages had high variance. One study did not specify the rodent strain used for obtaining MSCs, and three papers did not report the cellular passage. Five studies did not report donor genders.  Table 2 shows the LED parameters used in the articles. Studies showed a huge variety of irradiating primary MSCs using LEDs. Wavelengths were mostly within the red spectrum, ranging from 620 to 800 nm. One study considered irradiating cells at the blue wavelength (400 to 480 nm and another paper has applied irradiations in a broad spectrum of light, ranging from 400 to 800 nm). Many studies have not reported dimensions for flasks, dishes, or culture wells. In this regard, approximate growth surface areas were considered in the basic dimension guide (e.g., https://www.corning.com/catalog/cls/documents/application-notes/CLS-AN-209.pdf): 96-well plate (0.32 cm^2^); 24-well plate (1.9 cm^2^); 35 mm or 3.5 cm plate (9 cm^2^); and T75 bottles (75 cm^2^). Thus, the irradiance area varied from 0.32 to 75 cm^2^. Potency reached 0.848 to 900 mW, whereas the irradiance varied from 1.65 to 100 mW/cm^2^, appearing differently when distinct authors. Energy varied from 0.102 to 450 J, and radiant exposure reached 0.075 to 32 J/cm^2^. Irradiation time reported reached 10 to 3636 seconds, appearing differently when distinct authors. Single irradiation was most used, and the timeline varied between 1 and 28 days.

The main results are presented in Table 3. Most studies that assessed viability, proliferation, and differentiation showed that the cells responded positively to LED. These findings were accompanied by the increased metabolic potential of the cells, as illustrated by higher ATP content and mitochondrial activity. MSCs irradiated with red LED also had an increased secretion of nitric oxide (NO) and growth factors, such as the fibroblast growth factor (FGF), hepatocyte growth factor (HGF), and vascular endothelial growth factor (VEGF). There was no effect of LED on scratch, with one study reporting increased levels of genetic self-renewal markers. Only one study has reported an adverse effect of LED to increased DNA fragmentation. Moreover, the only study that has used blue wavelengths reported decreased viability using 3-(4,5-dimethyl-2-thiazolyl)-2,5-diphenyl-2H-tetrazolium bromide assay.

## 4. Discussion

Most of the therapeutic effects of MSCs are unsatisfactory because implanted cells have low engrafting and do not survive for a long time. In light of these undesirables, genetic approaches were used to improve survival, engraftment, proliferation, and differentiation of MSCs [43]. MSC preconditioning has been another strategy to enhance functionally and cellular resistance into the hostile tissue [43]. In this setting, the present study was carried out to systematically review the literature on the effectiveness of LED in optimizing the therapeutic potential of MSCs.

In respect to the irradiation approach, most studies have applied LED in the red band, varying between 620 and 800 nm [13, 44–51]. The red light was chosen because it has been well-reported to rise the proliferation rate of various MSCs lines [44, 45, 52]. Besides, the majority of LED applications on MSCs were performed with irradiances of up to 15 mW/cm^2^ and radiant exposure ranging from 0.075 to 4 J/cm^2^ [13, 44–47, 50, 51]. This LED exposure programming is very helpful to enhance cell proliferation [13, 44, 47, 53], showing to be in line with the findings described in Table 3. MSC proliferation was positively influenced by LED as analyzed by different assays. On the other hand, the only study that used LEDs at 420-480 nm wavelength reported a lower rate of proliferation compared to non-irradiated MSCs over the 28-day follow-up [54], indicating that blue light does not seem subtle to enhance MSCs.

The greatest cell proliferation was accompanied by a range of up-regulated cellular responses by red-light, including clonogenic potential [44, 51], osteogenic differentiation [44], gametogenesis [50], and endothelial as well as epithelial cell formation [46]. Only one study has reported that LED can induce negative effects as determined by MTT assay [54] and DNA fragmentation [48]. This may be a result of the high irradiance applied to the cells, which was much higher than most studies included in the review that has applied red-light. High irradiance has also been used by Lipovsky et al. [49] with increased cell proliferation; however, the authors have not examined any apoptosis marker. Finally, the number of irradiations varies between different studies (see Table 2). The better frequency of irradiations affecting MSCs has not yet been determined; however, there are data reporting that LED effect on the proliferation by single-dose irradiation is temporary, and multiple stimuli are necessary for the optimization of MSC growth [44, 55].

Biological mechanisms of light therapy are not yet fully understood, and many of the data come from investigations of low-level laser role on MSCs [56, 57]. It is difficult to assume a directly comparable effect of laser with LED because these light sources have some different features. Notwithstanding, in recent years, it has become that LED performs equally to medical lasers [58], with a suitable alternative without the laser's disadvantages such as heat production, narrow beam width, and high charges [59]. Mitochondrial respiratory chain activation is a well-described mechanism of red-light to increase ATP production which may accelerate mitosis [44, 60–62], and three studies in this review have reported increased proliferation associated with higher ATP level and mitochondrial activity [44, 45, 51]. The set of information available leads to proposing a complex signaling cascade, including singlet oxygen, mitogen-activated protein kinase/extracellular signal-regulated protein kinase, Janus kinase/signal transducer, and reactive oxygen species [44, 63–67].

In this review, two studies have reported a significant increase in VEGF, FGF, HGF, and NO content, respectively [45, 46, 49, 51]. These paracrine effects and immune regulatory functions of MSCs have been used to successfully treat a variety of tissue injury-related diseases [68, 69]. Moreover, the stimulation of angiogenesis is a very important effect of red-light to increase proliferation capacity [70, 71]. Thus, Szymanska et al. [72] reported an increased endothelial cell proliferation after light stimulation as possibly mediated by VEGF.

**Figure 3:**
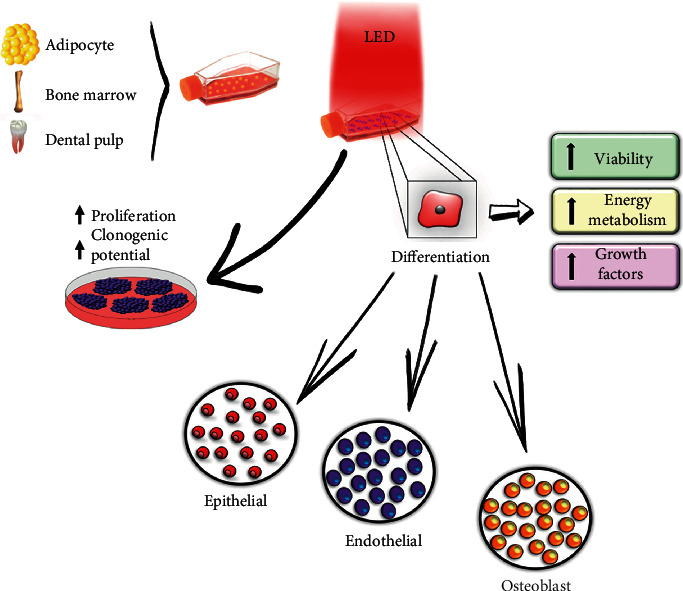
Main effects of LED irradiation to boost MSCs.

Therefore, the red LED may be associated with a prosurvival signal in the MSCs that added to the increased production of ATP and growth factor secretion would lead to greater cellular response to proliferation and differentiation. We summarize this network of red LED irradiation in Figure 3.

Although LED therapy could bring benefits in MSCs engineering, there was no unanimity regarding the source, nor the quantity and passage used in the experiments. Moreover, quality guideline criteria revealed that many of the included manuscripts had a high risk for detection, performance, and selection bias. These bias categories may be associated with problems in adhering to the study protocol, systematic differences introduced during the study, and lack of blinding results to study group or exposure level [42]. It is intended that a high risk of bias can potentially compromise the confidentiality of studies and influence the translation of findings in vitro to experiments using animals or clinical trials in the future.

Furthermore, after analyzing the studies in this review, it is important to quote some perspectives and limitations. The lack of studies available in irradiating MSCs with LEDs such as *in vitro* studies that mimic a hostile microenvironment, commonly found in transplantation sites, as well as *in vivo* experimentation and clinical trials possibly hindered the definition of a more effective irradiation protocol. Besides that, the lack of dosimetric parameters in studies that were not included in this review impossibilities the reproducibility and replicability of the results by other authors, also hindering determining the best parameter of irradiation.

## 5. Conclusion

Although the small number of studies limits the power of systematic review on photobiomodulation, evidence was found to suggest that red LED with a radiant exposure up to 7.2 J/cm^2^, which can be an effective approach to boost MSC therapy. Overall, MSCs exposed to LED have shown enhanced viability, proliferation, differentiation, cell metabolism, and secretion of angiogenic factors compared to nonirradiated MSCs.

## Figures and Tables

**Table 1:** Overall characteristics of selected studies for LED effects in mesenchymal stem cells.

References	Source	Species	Gender	Quantity/concentration^∗^	Experimental passage
[44]	Bone marrow	Rat	Female	100 cells/well (low density) 1,000 cells/well (high density)	P3
[45]	Tooth pulp	Rodent (NR)	NR	2.5 × 10^4^ cells/plate	P2-P3
[46]	Adipocyte	Human	NR	1.5 × 10^6^ cells	P5-P8
[13]	Bone marrow	Rat	NR	8 × 10^5^ cells/plate 3 × 10^3^ cells/well	NR
[47]	Umbilical cord	Human	Fresh neonatal	1 × 10^4^ cell/plate	P3-P5
[48]	Tooth pulp	Human	Male and female	10^4^ cells	P3-P6
[49]	Bone marrow	Rat	Male	1.3 × 10^6^ cells/cm^2^	NR
[50]	Umbilical cord	Human	NR	5 × 10^4^ cells/dish	P3-P5
[51]	Umbilical cord	Human	NR	5 × 10^3^ cells/well 5 × 10^5^ cells/well 1 × 10^6^ cells/well 1 × 10^5^ cells/well 200 cells/well	P3-P5
[54]	Gingiva	Human	NR	2 × 10^4^ cells/mL, 2.5 × 10^4^ cells/well	NR

NR: not reported. ^∗^Cell number varied according to each variable analyzed in the respective study.

**Table 2:** Irradiation LED protocol of selected studies.

References	Wavelength (nm)	Irradiation area (cm^2^)	Potency (mW)	Irradiance (mW/cm^2^)	Energy (J)	Radiant exposure (J/cm^2^)	Time of irradiation (sec)	Irradiation quantity	Timeline (days)
[44]	630	0.32	1.6^∗^ 4.8^∗^	5 15	0.64^∗^ 1.28^∗^ 0.64^∗^ 1.28^∗^	2 4 2 4	400 800 133 266	Once or multiple	28
[45]	653	9^#^	33.57^∗^	3.73	0.675^∗^ 1.341^∗^ 2.016^∗^ 3.024^∗^ 4.032^∗^	0.075 0.149 0.224 0.336 0.448	20 40 60 90 120	Once	15
[46]	660	1.9^#^	19^∗^	10	1.9^∗^ 3.8^∗^ 11.4^∗^ 17.1^∗^	1 3 6 9	600	Once	14
[13]	620	0.32^#^	2.13^∗^	6.67	0.32^∗^ 0.64^∗^ 0.128^∗^	1 2 4	150 300 600	Multiple	21
[47]	625	0.32^#^	0.848^∗^	2.65	0.102^∗^ 0.204^∗^ 0.305^∗^	0.318 0.636 0.954	120 240 360	Once	14
[48]	630	1.9^#^	70.3^∗^	37	3.8^∗^ 7.6^∗^ 15.2^∗^ 30.4^∗^ 60.8^∗^	2 4 8 16 32	54 107 214 428 856	Once	1
[49]	400-800	9^#^	360^∗^	40	21.6^∗^ 43.2^∗^ 64.8^∗^	2.4 4.8 7.2	60 120 180	Once	**7**
[50]	625	9^#^	47.7^∗^	5.3	17.1^∗^	1.9	360	Once	21
[51]	633	75^#^	534^∗^ 123.75^∗^	1.65 7.12	22.5^∗^ 75^∗^ 225^∗^ 450^∗^ 22.5^∗^ 75^∗^ 225^∗^ 450^∗^	0.3 1 2 6 0.3 1 3 6	182 606 1818 3636 42 140 421 843	Once	10
[54]	420-480	9^#^	900^∗^	100	9^∗^ 18^∗^ 36^∗^ 54^∗^	1 2 4 6	10 20 40 60	Multiple	28

^∗^Missing parameters have been calculated. ^#^Data were calculated on the culture flasks/dishes/wells areas.

**Table 3:** Main effects of LED irradiation on mesenchymal stem cells.

References	Viability	Proliferation	DNA damage	Differentiation	Metabolism	Secretome	Senescence	Scratch
[44]	NE	↑ Liu's staining (multiple LED applications: 5 and 15 mW/cm^2^; 2 and 4 J/cm^2^) ↑CFU-F (multiple LED applications: 15 mW/cm^2^; 4 J/cm^2^)	NE	↑ Osteogenic (ALP activity; osteocalcin expression; (multiple LED applications: 15 mW/cm^2^; 4 J/cm^2^)	↑ ATP (single LED application: 15 mW/cm^2^; 4 J/cm^2^)	NE	NE	NE
[45]	NE	↑ BrdU	NE	NE	↑ Mitochondrial activity ↑ ATP	↑ NO (nitrite)	NE	NE
[46]	NE	NE	NE	*↑* Endothelial cells (CD31, CD34, and KDR) *↑* Epithelial cells (cytokeratin)	NE	↑ FGF, HGF and VEGF (6 J/cm^2^)	NE	NE
[13]	NE	↑ WST-8 (1, 2 and 4 J/cm^2^) ↑ EdU staining (2 J/cm^2^)	NE	= Osteogenic (ALP activity; Alp1, Bglap, Col1*α*1, Runx2, and gene expression) = Mineral nodule formation (Von Kossa staining)	NE	NE	NE	NE
[47]	= Trypan blue	↑ WST-1 (0.954 J/cm^2^) ↑ Hoechst staining = CFU-F	NE	NE	NE	NE	NE	=
[48]	NE	NE	↑ Fragmented DNA (4 and 32 J/cm^2^)	NE	═ Mitochondrial membrane potential	NE	═ *β*-Galactosidase staining	NE
[49]	NE	↑ Cell counts (2.4, 4.8, and 7.2 J/cm^2^)	NE	NE	↑ ROS	↑ NO (nitrite) (4.8 and 7.2 J/cm^2^)	NE	NE
[50]	NE	NE	NE	↑ Gametogenic (gene/protein expression: DAZL and SCP3)	NE	NE	NE	NE
[51]	NE	↑ WST-1 and ↑ CFU-F (7.12 mW/cm^2^: 1 J/cm^2^)	NE	= Adipogenic (red oil; gene expression: PPAR*γ*, LPL) = Osteogenic (Alizarin red S; gene expression: ALP, Bglap)	↑ Mitochondrial activity (7.12 mW/cm^2^: 1 J/cm^2^)	↑ FGF and VEGF	↑ Self-renewal (genes: NANOG, OCT4 and SOX2; 7.12 mW/cm^2^: 1 J/cm^2^)	NE
[54]	NE	↓ MTT (1, 2, 4 and J/cm^2^)	NE	↑ Osteogenic (ALP activity; gene expression: gene type I, osteocalcin and Runx2) ↑ Mineralization (Alizarin red oil)	NE	NE	NE	NE

NE: not evaluated; ↑: increase; ↓: reduction; =: not changed. ALP: alkaline phosphatase; ATP: adenosine 5´-triphosphate; Bglap: osteocalcin; BrdU: 5-bromo-2-deoxy-uridine; CFU-F: colony-forming unit fibroblasts; Col1*α*1: collagen type I; FGF: fibroblast growth factor; HGF: hepatocyte growth factor; LPL: lipoprotein lipase; MTT: 3-(4:5-dimethyl-2-thiazolyl)-2:5-diphenyl-2H-tetrazolium bromide; NANOG: nanog homeobox; NO: nitric oxide; PPAR*γ*: peroxisome proliferator-activated receptor gamma; OCT4: octamer-binding transcription factor 4; ROS: reactive oxygen species (nonfluorescent marking 2′:7′-dichlorofluorescin diacetate); Runx2: runt-related transcription factor 2; SOX2: sex determining region Y-box; VEGF: vascular endothelial growth factor; WST-8: water-soluble tetrazolium.
